# Real-Life Language Use Across Different Interlocutors: A Naturalistic Observation Study of Adults Varying in Age

**DOI:** 10.3389/fpsyg.2019.01412

**Published:** 2019-06-25

**Authors:** Minxia Luo, Megan L. Robbins, Mike Martin, Burcu Demiray

**Affiliations:** ^1^Department of Psychology, University of Zurich, Zurich, Switzerland; ^2^University Research Priority Program “Dynamics of Healthy Aging”, University of Zurich, Zurich, Switzerland; ^3^Department of Psychology, University of California, Riverside, Riverside, CA, United States

**Keywords:** Electronically Activated Recorder, cognitive aging, conversations, social context, audience design, corpus linguistics, vocabulary richness, grammatical complexity

## Abstract

Amid the growing interest in studying language use in real life, this study, for the first time, examined age effects on real-life language use, as well as within-person variations across different interlocutors. We examined speech samples collected via the Electronically Activated Recorder (i.e., portable audio recorder that periodically records ambient sounds) for a larger project. This existing dataset included more than 18,000 sound snippets (50-s long) from 53 American couples (breast cancer patients and their spouses; aged 24 to 94 years) in their natural environments. Sound snippets that included participant speech were coded for different interlocutors and given scores on three linguistic measures that are associated with age-related cognitive changes: usage of unique words, usage of uncommon words, and grammatical complexity. Multilevel models showed that there were no age effects on the three linguistic measures when interlocutors were not taken into account. We found that interlocutors influenced usage of unique words and grammatical complexity. More specifically, compared to talking with their spouse, participants used fewer unique words with children and friends; and used simpler grammatical structures with children, strangers, and in multiparty conversations. Next, we found that interlocutors influenced the associations between age and language use. More specifically, young adults used more unique words and more uncommon words with children than older adults. They used more uncommon words with friends and uttered more complex grammatical structures with strangers than older adults. Our results offer preliminary evidence for a new perspective to understand real-life language use: focusing not only on individual characteristics (i.e., age), but also context (i.e., interlocutors). This perspective should be useful to researchers who are interested in collecting “big data” and understanding cognitive activities in real life.

## Introduction

Language use in old age has been an active research area since early experimental work on cognitive aging (e.g., Kemper and Anagnopoulos, 1989; Burke and Shafto, 2008). Furthermore, there has been a growing interest in extending the examination of age effects on language use to real life (Horton et al., 2010; Gahl et al., 2014). In theory, behavior is determined by both individuals’ characteristics and contexts (Lewin, 1951; Lawton, 1983; Diehl and Willis, 2003; Martin and Moor, 2012; WHO, 2015). In other words, with given abilities, individuals’ behaviors should vary depending on contextual factors. However, most cognitive aging studies have depicted language use as primarily determined by age-related cognitive changes and neglected context (e.g., Horton et al., 2010). Only recently, some researchers started to investigate the effects of interlocutors, as one aspect of context, on language use, in addition to the effects of cognitive aging (Meylan and Gahl, 2014; Moscoso del Prado Martín, 2016). However, these studies treated interlocutor effects as control variables, without explicitly identifying contextual factors in their theoretical frameworks. Moreover, most studies that examined contextual effects have focused on between-person differences, which are limited in inferring how the same speaker changed their language across different contexts (Hamaker, 2012). Furthermore, they relied on speech samples from language production tasks in telephone conversations, which may not be representative of language use that occurs naturally in everyday life.

This study, for the first time, examined real-life language use by utilizing a naturalistic observation method and focusing on age and within-person variations across different interlocutors. “Real-life language use” in our study refers to language use that naturally occurs in everyday life in contrast to language that is produced in language production tasks in the laboratory or in telephone conversations (e.g., Burke and Shafto, 2008; Horton et al., 2010). The Electronically Activated Recorder (EAR; Mehl et al., 2001), a digital recorder which periodically and unobtrusively captures ambient sounds and speech, was used to collect speech samples in everyday life. We investigated usage of unique words, uncommon words, and grammatical complexity that have been found to be associated with age-related cognitive changes (e.g., Cheung and Kemper, 1992; Horton et al., 2010). We hypothesized that, in real life, these linguistic measures are not only determined by age, but also by interlocutors and the interaction between age and interlocutors. Our first goal was to examine age effects in these dimensions of language use in speakers’ natural environments. Our second goal was to study whether and how different interlocutors influenced language use in real life. Finally, our third goal was to investigate whether interlocutors influenced the relation between age and language use. As this is a first attempt, our results provide preliminary evidence and should be bolstered with future research. However, our study offers a new perspective to examine real-life language use, focusing not only on individual characteristics (i.e., age), but also on context (i.e., interlocutors).

### Effects of Age and Interlocutors on the Usage of Unique Words, Uncommon Words, and Grammatical Complexity

With the perspective of considering both individual characteristics and contextual factors in understanding real-life behavior (e.g., WHO, 2015), we reviewed past cognitive aging studies on the usage of unique words, uncommon words, and grammatical complexity in the following section.

#### Usage of Unique Words

Usage of unique words (i.e., the number of different words relative to the number of total words produced), represents the vocabulary size of an individual in language production (Burke and Shafto, 2008). Age has been found to be positively associated with the usage of unique words in laboratory monolog tasks (Kemper and Sumner, 2001; Kemper et al., 2010). This finding is in line with the commonly observed positive relation between scores in vocabulary tests and age (Cheung and Kemper, 1992; Verhaeghen, 2003), and with accumulated vocabulary knowledge through lifelong experience (Ramscar et al., 2014). Aiming to increase ecological validity, some studies examined age-related changes in language use during telephone conversations and found that older adults used more unique words than young adults (Horton et al., 2010; Meylan and Gahl, 2014; Moscoso del Prado Martín, 2016). Additionally, effects of interlocutors were examined. Meylan and Gahl (2014) found that participants (aged 17 to 68) used more unique words while talking with older people and men than young people and women. As the study showed that older and male participants used more diverse vocabulary than young and female participants, the authors suggested that interlocutor effects can be related to speakers matching their language to different interlocutors.

#### Usage of Uncommon Words

Producing uncommon words (i.e., words that are infrequently used) is another indicator of having a large vocabulary (Burke and Shafto, 2008). Older adults used more uncommon words than young adults in laboratory description tasks (Kavé et al., 2009). Accumulated vocabulary knowledge in aging enables older adults to have more words at their disposal (Verhaeghen, 2003; Ramscar et al., 2014). However, in telephone conversations, Horton et al. (2010) found no age differences in the usage of uncommon words. It is unclear why age effects observed in the laboratory were not found in telephone conversations. In general, empirical evidence on the usage of uncommon words in real life is insufficient. Furthermore, interlocutors, who may influence the usage of uncommon words, have not been examined.

#### Grammatical Complexity (Clauses per Sentence)

The number of clauses (i.e., sub-sentences that consist of a subject and a verb) in a sentence indicates how complex the grammatical structure of a sentence is (Burke and Shafto, 2008). In laboratory monolog tasks, the number of clauses per sentence declined with age (Cheung and Kemper, 1992; Kemper et al., 2010). This was explained with older adults having reduced working memory capacity, which affected their ability to generate complex grammatical elements (Cheung and Kemper, 1992). However, in telephone conversations, the number of clauses per sentence was unrelated to age (Horton et al., 2010). The authors suggested that age effects were attenuated in the real world, where older adults can modify their language to achieve their communication goals. Furthermore, they admitted that their study did not take into account the potential impact of interlocutors. In response to the limitations of this study, Moscoso del Prado Martín (2016) examined the effects of interlocutors and found that participants who talked to men used more complex grammar than those who talked to women. Although the author did not discuss why more complex grammar was used with men than women, this finding hinted at potential effects of interlocutors.

In sum, older adults generally used more unique words, more uncommon words, and simpler grammatical structures than young adults in laboratory monolog tasks. In addition to speaker’s verbal ability, interactions between interlocutors in conversations may as well influence speakers’ language use (Clark, 1996; Linell, 1998). Researchers recently started to take into account interlocutor effects in language use, but they did not explicitly point out the importance of context in their theoretical frameworks (Meylan and Gahl, 2014; Moscoso del Prado Martín, 2016). Furthermore, the findings of interlocutor effects came from studies with between-person designs, which are limited in inferring how the same speaker altered their language with different interlocutors (Hamaker, 2012). Moreover, studies relying on one-off speech production tasks in the laboratory or via telephone conversations may not be representative of naturally occurring language use in real life. Thus, it is important to investigate both age effects on language use, and within-person variations in language use across different interlocutors. Additionally, it is important to examine real-life language data to complement existing studies with laboratory and telephone speech samples.

### Audience Design, Age, and Language Use

Interlocutors have been conceptualized as an important determinant of language use in audience design research. Evidence in this line of research can offer hints to explain the mechanism of interlocutor effects on language use. Audience design refers to the phenomenon when speakers shift their utterances primarily in response to their audience (Clark and Brennan, 1991; Schober and Brennan, 2003). This phenomenon is identified when speakers vary their language across different listeners on the basis of common ground, e.g., knowledge or beliefs about the audience, which can be the background, relationship with the speaker, and needs in comprehension (Krauss, 1987; Horton and Gerrig, 2002). For example, in laboratory recall tasks, participants spoke to attentive listeners in greater detail than to distracted listeners (Pasupathi et al., 1998). When recalling a story, participants spoke more about their subjective evaluations of the story with peer participants than with experimenters (Hyman, 1994). In a laboratory cueing game, due to mutual understanding among familiar interlocutors, participants needed fewer words to help their spouse than strangers to guess the target information (Rauers et al., 2011). In a multiparty communication task, speakers produced more words to help the least knowledgeable audience in the group to understand the communication content (Yoon and Brown-Schmidt, 2017).

Furthermore, although inconclusive, past studies have shown that age influenced the effects of interlocutors on language use (i.e., Age × Interlocutor interaction). For example, in communication tasks, whereas young adults produced fewer words with familiar interlocutors than unfamiliar interlocutors, older speakers had little variation in their language use (Horton and Spieler, 2007). The authors explained these age differences in language variation as older adults having difficulties in accessing memory representations of the interlocutors. However, in a recent study, both young and older adults produced fewer words with familiar interlocutors than unfamiliar interlocutors, although young adults produced even fewer words than older adults (Yoon and Stine-Morrow, 2019). The authors explained that the communication between participants and interlocutors in their study was interactive, in contrast to the one-way communication in Horton and Spieler (2007). In turn, interlocutors’ feedback may have provided the older adults with more contextual cues. On the contrary, when retelling a story, although both young and older adults used more elaborations, repetitive words, and simplified speech (i.e., lower scores in a complexity index representing fewer syllables per word and fewer number of words per sentence) with a child than with an experimenter, older adults used even simpler speech than young adults (Adams et al., 2002). These findings were interpreted in the context of goals in aging: Older adults prioritized emotionally meaningful life goals and thus simplified their language to transmit information to children (Carstensen et al., 1999).

In sum, interlocutors have been an important determinant of language use in audience design research. Additionally, despite the mixed results on age effects, this research has shown Age × Interlocutor interaction effects on language use. Although most of the interlocutor effects reviewed in this section were not directly related to the usage of unique words, uncommon words, and grammatical complexity, they offer potential explanations for interlocutor effects and highlight the value of examining interlocutor effects on language use in the context of aging.

## The Current Study

This study is part of a larger project on couples coping with breast cancer conducted at the University of Arizona and Arizona Cancer Center. We used the existing EAR dataset from this project to examine real-life language use for the first time in the cognitive aging literature. The EAR method was used to collect samples of everyday conversations and to examine communication processes of couples coping with cancer in their natural environments (Robbins et al., 2014; Karan et al., 2017). With high compliance and low obtrusiveness, the EAR has been widely used to observe real-life language use (Mehl and Pennebaker, 2003; Mehl, 2017), but no EAR studies to date have focused on cognitive aging and language. The intensive and repeated sampling approach of the EAR captures multiple observations from each participant and, thus, allows us to analyze within-person variations in language use across interlocutors in real life. Considering that it requires excessive amounts of time and effort to process EAR audio files, we used this existing EAR dataset as a first step to examine both age effects and within-person variations in language use. Since this dataset included patients with breast cancer and may not be representative of the general population, we controlled for the effects of participants’ characteristics.

The first goal of our study was to investigate age effects on the usage of unique words, uncommon words, and grammatical complexity. Given that there were only a few studies that have analyzed age effects on the three dimensions of language use in telephone conversations (e.g., Horton et al., 2010), we formed our hypotheses on the basis of laboratory findings (e.g., Cheung and Kemper, 1992). Thus, we expected older adults to use more unique words, more uncommon words, and simpler grammatical structures than young adults in real life, regardless of whom the speakers talked with.

The second goal of our study was to examine whether and how interlocutors influenced real-life language use. When we found a significant interlocutor effect, we considered it in support of our perspective that interlocutor effects should be examined in real-life language use in cognitive aging studies. We focused on the effects of different types of interlocutors on language use and formed our hypotheses by referring to the audience design research. First, when talking with children, participants used more repetitive words and simpler speech (i.e., lower scores in a complexity index representing fewer syllables per word and fewer number of words per sentence; Adams et al., 2002). Thus, we hypothesized that participants would produce fewer unique words, fewer uncommon words, and simpler grammatical structures with children than with the spouse. Second, participants used fewer words to communicate information to familiar than unfamiliar interlocutors (Horton and Spieler, 2007; Rauers et al., 2011; Yoon and Brown-Schmidt, 2017; Yoon and Stine-Morrow, 2019). Given that interlocutors with different levels of familiarity had effects on language use (i.e., word count), we examined whether the usage of unique words, uncommon words, and grammatical complexity differed across various interlocutors that may exist in real life, i.e., the spouse, friends, family members, strangers, and in multiparty conversations.

The third goal of our study was to examine whether interlocutors influenced the relation between age and language use (i.e., Age × Interlocutor interaction). When an Age × Interlocutor interaction was shown, we considered it offered support for our anticipation that age effects on language use would be influenced by interlocutors. Adams et al. (2002) found that older adults had lower scores in a complexity index than young adults, which represented fewer syllables per word and fewer number of words per sentence, with children. Thus, we expected that older participants would use fewer uncommon words and simpler grammatical structures than young participants while talking with children. We also explored whether there were age differences in the usage of unique words with children. Furthermore, studies have shown that while young adults reduced number of words with familiar interlocutors (in comparison to unfamiliar ones), older adults reduced fewer number of words than young adults or did not change (Horton and Spieler, 2007; Yoon and Stine-Morrow, 2019). Thus, we explored whether there were any age differences in the usage of unique words, uncommon words, and grammatical complexity across the interlocutors of spouse, friends, family members, strangers, and in multiparty conversations. Finally, we controlled for the possible effects of other individual characteristics, including education, role (i.e., patients, partners), depression scores, and patients’ illness stage.

## Materials and Methods

### Participants

The sample included more than 18,000 sound files collected from 104 American adults (i.e., 53 couples with one breast cancer patient and one spouse missing). Their age range was 24 to 94 years (*M* = 57.78, *SD* = 14.37). Among the 53 couples (eight same-sex), 60 participants were female (58%). Years of education ranged from nine to 21 (*M* = 15.34, *SD* = 2.48). Patients’ cancer stage ranged from 0 to 4 (Stage 0: 3.8%, Stage I: 30.8%, Stage II: 26.9%, Stage III: 23.1%, Stage IV: 7.7%, Unknown: 7.7%). The average score on the Center for Epidemiologic Studies Depression Scale (CES-D, Radloff, 1977) was 11.27 (*SD* = 8.50, Range: 0–37). A score of 16 or more on the CES-D is typically considered “depressed.” All participants were primarily English speakers. Eighty-two percent of participants were Caucasian (*n* = 85), 13% Latino (*n* = 14), 2% African American (*n* = 2), 2% Asian (*n* = 2), and 1% American Indian (*n* = 1). The couples were living together in a romantic relationship, with relationship length ranging from 0.4 to 61.7 years (*M* = 23.1, *SD* = 15.8). Each couple received $150 for their participation.

### Procedures

Participants were invited to the laboratory on a Friday afternoon to complete questionnaires as part of the larger study, and were then provided with an introduction to the EAR protocol. They were instructed to wear the EAR as much as possible. They were informed that the EAR would record multiple 50 s of ambient sounds to capture approximately 10% of their waking hours. They were notified that the sound files would be recorded without their awareness and that they should proceed with their normal everyday life. They were informed that the EAR would cease recording during their sleeping hours (i.e., starting 30 min after they indicated they typically go to sleep). They were informed that they would have an opportunity to review all audio recordings prior to anyone else listening to them. Afterward, they wore the EAR over the weekend. Typically, on a Monday after the weekend, participants went back to the laboratory to return the EAR and to complete questionnaires on demographic and medical information. They were given a password-protected CD containing their sound files to review. All study procedures were approved by the Institutional Review Board at the University of Arizona.

#### EAR

The EAR software was programmed on an HP iPAQ 100 handheld computer. It was programmed to record 50 s of ambient sounds every 9 min. The device was housed in a protective case affixed to participants’ waistlines and an external microphone (Olympus ME-15) was attached to participants’ lapels. The EAR was programmed to not record for 6 h during the participants’ predefined sleep hours. The EAR recorded participants’ waking days, from the time the participant received the device until they went to sleep on Sunday night. About 176 (*SD* = 57) 50-s sound files were collected per participant across a weekend.

### Linguistic Measures

All utterances of the participants captured by the EAR were transcribed. A research assistant created the transcripts, which were then checked and corrected by a second research assistant. Lexical fillers, such as “you know,” “well,” and “yeah,” and non-lexical fillers, such as “umm” and “uh” were retained in the transcripts. The utterances from the interlocutors or bystanders were not transcribed. The utterances that were not clear to coders were transcribed as “xxxx.” We used the TreeTagger (Schmid, 1994) via the R package of “koRpus” version 0.10-2 (Michalke, 2018) to identify each word according to its grammatical class (e.g., a noun, a verb, an adjective), a process called *part-of-speech tagging*. We also turned each word to its lemma form, a process called *lemmatization*. For example, we turned “go,” “went,” and “gone” to the lemma form of “go,” and transformed “apple” and “apples” to the lemma form of “apple.” Afterward, we calculated the following linguistic measures.

#### The Usage of Unique Words: Entropy

The usage of unique words was represented with Shannon entropy measure (Shannon, 1948),

$$H\;[L]=-\sum_{i\in L}p(i)\log p(i)$$

where the *p*(*i*) is the probabilities of a given word. We categorized each word according to their lemma form and part-of-speech tags. We, then, calculated the entropy scores in each sound file with the Chao–Shen estimator (Chao and Shen, 2003; according to Moscoso del Prado Martín, 2016) using the R package of “entropy,” version 1.2.1 (Hausser and Strimmer, 2014)^1^. For example, “I like apple and orange.” has the score of 5.03, whereas “I like apple and apple.” has a score of 3.29. Lower scores of entropy indicate more repetitiveness and thus lower usage of unique words.

#### The Usage of Uncommon Words: The Average Frequency of Nouns

The average frequency of nouns has been used as an indicator of the usage of uncommon words in linguistics studies (e.g., Horton et al., 2010; Kavé et al., 2009). Lower frequency of a word indicates that the word is less commonly used. Based on the lemma and the part-of-speech tags, we extracted the words that had been tagged as either a noun (NN) or a plural noun (NNS). We then used the American National Corpus spoken lemma-form database (3,862,171 words; Reppen et al., 2005) to obtain the frequency of each noun in its lemma form. The frequency of each noun was represented as frequency per million in the database. Finally, we calculated the average score of the frequency of nouns in each sound file. Higher average frequency of nouns indicates less usage of uncommon words.

#### Grammatical Complexity: Clauses per Sentence

Clauses per sentence is the ratio of clauses to sentences and represents grammatical complexity (Kemper et al., 2010; Horton et al., 2010). A clause is defined as a structure with a subject and a finite verb. A sentence is a group of words delimited with one of the following punctuation marks that signal the end of a sentence: period, question mark, exclamation mark, quotation mark, or ellipsis (Lu, 2010). The score of clauses per sentence in each 50-s sound file was computed with the open-source Python code of Syntactic Complexity Analyzer (Lu, 2010).

### EAR Coding

#### Interlocutor(s)

Every sound file has been manually coded for interlocutors(s): spouse, child(ren), family member(s), friend(s), stranger(s), self, pet(s), and unknown^2^. Trained coders coded the role of each interlocutor based on conversation topics, pitch of the voice, ambient sounds, and adjacent EAR sound files. All coding categories were dichotomous, indicating the presence (1) or absence (0) of an interlocutor within a sound file. As the categories of interlocutors were not mutually exclusive, we computed an additional category of “multiparty conversations” to indicate that more than one type of interlocutor was present in a given sound file. All sound files were double-coded by two independent research assistants and the two sets of coding were averaged across each participant’s total set of coded sound files. One-way random effects intraclass correlations (ICC[1;2]) indicating inter-coder reliability ranged from 0.51 to 0.93^3^.

## Results

### Preliminary Analyses

We had a total of 6,672 sound files which included participant speech (more than 223,000 spoken words; about 46% of all sound files collected). We excluded 139 sound files coded as talking with one’s self, 267 sound files coded as talking with pet(s), and 121 sound files coded exclusively as talking with unknown people, as we were interested in conversations and specific interlocutors. We used the remaining 6,147 sound files for analyses (Range: 5–159 per participant, *M* = 59.11, *SD* = 29.96).

Among the 6,147 sound files, averaging across participants, the most frequently observed interlocutor was the spouse (*M* = 56.9%, *SD* = 26.4%), followed by “multiparty” (*M* = 23.2%, *SD* = 20.2%), friend(s) (*M* = 11.1%, SD = 14.8%), child(ren) (*M* = 4.5%, *SD* = 9.7%), family member(s) (*M* = 3.0%, *SD* = 7.8%), and stranger(s) (*M* = 1.5%, *SD* = 3.1%). Additionally, the category of “multiparty” included 93% of the time the spouse, 48% friends, 33% children, 22% family members, 9% strangers, and 4% unknown. Table 1 depicts the correlation matrix for the percentage of time spent talking with different interlocutors and participants’ characteristics (e.g., age, education). Older age was associated with higher percentage of time spent with the spouse, but lower percentage of time spent with children.

**Table 1:** Intercorrelations between participants’ characteristics and percentage of time spent with different interlocutors.

Variable	1	2	3	4	5	6	7	8	9	10	11	12
1. Age	1											
2. Gender(0 = women; 1 = men)	0.13	1										
3. Role (0 = patient; 1 = partner)	0.09	0.86^*^	1									
4. Years of education	0.06	-0.04	0.11	1								
5. Patients’ cancer stage (0-4)	-0.14	-0.01	-0.01	0.11	1							
6. CES-D scores	0.10	0.02	-0.07	-0.6	0.04	1						
7. % talking with Spouse	0.26^*^	0.06	0.10	-0.14	-0.40^*^	-0.07	1					
8. % talking with Child(ren)	-0.46^*^	-0.01	0.01	0.11	0.34^*^	-0.02	-0.53^*^	1				
9. % talking with Family member(s)	-0.01	0.01	-0.04	-0.02	0.15	-0.08	-0.29^*^	0.09	1			
10. % talking with Friend(s)	-0.01	-0.05	-0.04	-0.05	0.14	-0.01	-0.31^*^	-0.09	-0.07	1		
11. % talking with Stranger(s)	0.02	0.09	0.02	0.03	-0.02	0.12	-0.03	-0.10	0.12	-0.12	1	
12. % talking in Multiparty	-0.11	-0.05	-0.09	0.16	0.20	0.13	-0.71^*^	0.26^*^	-0.02	-0.24^*^	-0.03	1


*CES-D, Center for epidemiologic studies depression scale (Radloff, 1977); Multiparty, Multiparty conversations. ^∗^*p* < 0.05.*

**FIGURE 1:**
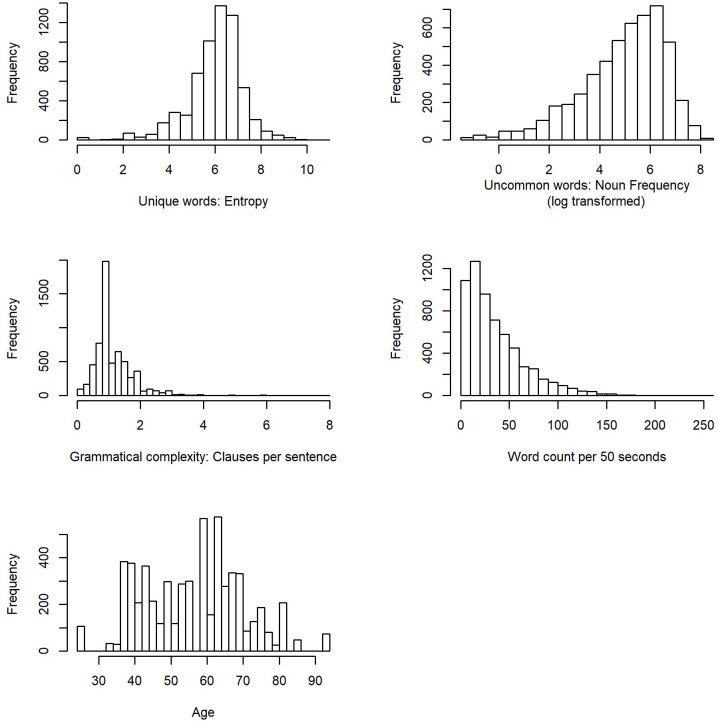
Histograms of the linguistic measures and age.

**FIGURE 2:**
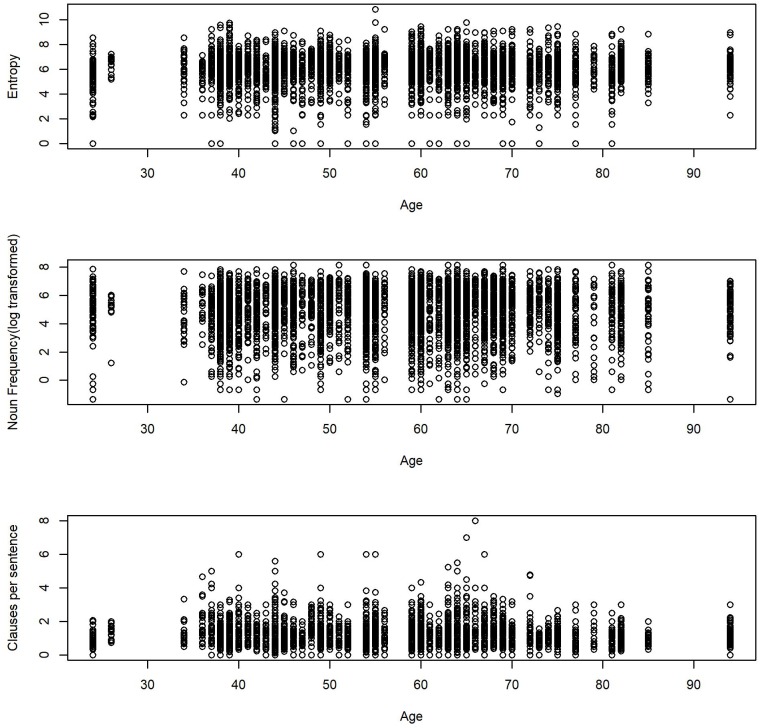
Scatter plots for the relations between linguistic variables and age.

The average score of entropy in each sound file was 6.01 (*SD* = 1.2, Mdn = 6.2, Range: 0.0–10.84). The nouns in each sound file appeared on average 344.45 (*SD* = 426.35, Mdn = 190.22, Range: 0.26–3461.52) times per million words in the American National Corpus database. The average number of clauses per sentence was 1.20 (*SD* = 0.6, Mdn = 1, Range: 0–8)^4^. Finally, participants uttered, on average, 36.5 words per 50 s (*SD* = 30.2, Mdn = 28, Range: 1–258). Figure 1 shows the histograms of the linguistic measures and age. Table 2 displays the correlation matrix for the linguistic measures and age. Figure 2 shows the scatter plots for the relations between each outcome linguistic measure and age.

**Table 2:** Correlation matrix for the linguistic measures and age.

Variable	Entropy	Frequency of Nouns (log)	Clauses per sentence	W.C. per 50s	Age
Entropy	1				
Frequency of Nouns (log)	0.18^∗∗∗^	1			
Clauses per sentence	0.19^∗∗∗^	0.15^∗∗∗^	1		
W.C. per 50 s	0.33^∗∗∗^	0.27^∗∗∗^	0.48^∗∗∗^	1	
Age	0.03	0.01	-0.01	-0.03^∗^	1


*W.C. per 50 s, Word count per 50 s. ^∗^*p* < 0.05, ^∗∗∗^*p* < 0.001.*

### Analytical Approach

The dyadic data in this study had a hierarchical structure: sound files nested within individuals, which were nested within the couples. The dyad members in the couples in our study are distinguished from one another by “role” (i.e., patient and partner). In the distinguishable dyads context, it is not suitable to estimate the data with three-level models: There is no random variability at the person level in distinguishable dyads, while multilevel models assumed random variability at each level of analysis (Kenny and Kashy, 2011; Bolger and Laurenceau, 2013). Thus, we followed a two-step procedure to analyze the dyadic data. First, we estimated two-intercept models to detect potentially different effects for patients and their partners. This model treated the three levels of dyadic data as two levels of random variation. In the lower level, the data of a patient and a partner of a dyad were separately fitted into two equations and then the between-dyad differences were represented in the upper level (Bolger and Laurenceau, 2013). More specifically, we estimated separate models for unique words, uncommon words, and clauses per sentence. In level-one equations, we examined interlocutor effects on language use. The spouse was the reference group for comparisons across interlocutors. In level two, we explained the random intercepts of the level-one equations with age^5^. Additionally, we added Age × Interlocutor interactions to the above estimated models to examine whether age effects in language use were influenced by interlocutors. Second, the two-intercept models cannot statistically test whether effects for patients and partners differed significantly. In order to test the potential differences between patients and partners, we estimated single-entry multilevel models with the same fixed and random effects as in the two-intercept models. Additionally, we added interactions between predictors and a variable representing patients versus partners (Kenny et al., 2006; Kenny and Kashy, 2011). This variable was effect coded (1 = patient, -1 = partner) and called “role” in the following sections. If an interaction between a predictor and “role” in a single-entry multilevel model was significant, it indicated that there were significant differences between patients and partners in the predictor.

In each model, we decomposed each dummy-coded interlocutor predictor into how the predictor varied on average from participant to participant (i.e., between-person variance) and how the predictor varied within each participant over time (i.e., within-person variance; Bolger and Laurenceau, 2013). We treated the within-person interlocutor effects as our predictors. Furthermore, we controlled for years of education, depression scores, patients’ illness stage, and “word count per 50 s.” The continuous fixed-effect variables were centered at the grand mean for ease of interpretation. Finally, we log transformed the variable of average frequency of nouns, as it was skewed.

We used the R package “lme4,” version 1.1-17 (Bates et al., 2018) in R Version 3.5.0 (R Core Team, 2018) to estimate the models and the 95% confidence intervals (CI). We additionally calculated *p*-values with R package “lmerTest” version 3.0-1 (Kuznetsova et al., 2018) and considered *p* < 0.05 as significant. We also estimated pseudo *R*-squared values as the percentage of variance explained after accounting for fixed effects of the fitted models with R package “MuMIn,” version 1.40.4 (Bartoñ, 2018).

### Major Analyses

Our first goal was to examine age effects in the usage of unique words, uncommon words, and grammatical complexity. Our second goal was to examine within-person variations in language use across different interlocutors. For these goals, we tested the main effects of age and interlocutors in language use, which are presented in Table 3. Our third goal was to inspect whether interlocutors influenced age effects in language use, therefore we added Age × Within-Person Interlocutor interactions to the analyses. Because their effects were not significant, we dropped depression scores and patients’ illness stage from all the models and we dropped education from the model of unique words and uncommon words.

**Table 3:** Two-intercept models on language use.

	Model 1: Entropy		Model 2: Frequency of nouns		Model 3: Clauses per sentence	
			
Parameter	Est.	95% CI	Exp.(Est.)(100%)	95% CI	Est.	95% CI
**Fixed effects**						
**Intercept**						
Patient	6.00^∗∗∗^	[5.93, 6.08]	128.16^∗∗∗^	[119.41, 137.41]	1.21^∗∗∗^	[1.16, 1.27]
Partner	6.04^∗∗∗^	[5.98, 6.10]	124.77^∗∗∗^	[115.38, 134.80]	1.18^∗∗∗^	[1.13, 1.22]
**Age**						
Patient	0.00	[0.00, 0.01]	1.00	[1.00, 1.01]	0.00	[0.00, 0.00]
Partner	0.00	[0.00, 0.01]	1.00	[1.00, 1.01]	0.00	[0.00, 0.00]
**WP Interlocutors:**						
**Child(ren)**						
Patient	-0.26^∗^	[-0.47, -0.06]	0.95	[0.69, 1.32]	-0.19^∗∗∗^	[-0.29, -0.09]
Partner	-0.01	[-0.22, 0.19]	0.76	[0.55, 1.04]	-0.05	[-0.14, 0.05]
**Family member(s)**						
Patient	0.07	[-0.18, 0.31]	0.97	[0.65, 1.43]	-0.11	[-0.23, 0.01]
Partner	-0.06	[-0.37, 0.24]	0.91	[0.56, 1.49]	-0.02	[-0.17, 0.12]
**Friend(s)**						
Patient	-0.24^∗∗^	[-0.39, -0.10]	1.04	[0.83, 1.31]	-0.06	[-0.13, 0.01]
Partner	0.04	[-0.12, 0.20]	1.02	[0.80, 1.30]	-0.07	[-0.15, 0.00]
**Stranger(s)**						
Patient	-0.38	[-0.77, 0.01]	0.91	[0.49, 1.68]	-0.23^∗^	[-0.42, -0.05]
Partner	-0.21	[-0.55, 0.13]	0.66	[0.39, 1.12]	-0.06	[-0.22, 0.10]
**Multiparty**						
Patient	-0.05	[-0.16, 0.06]	1.10	[0.93, 1.31]	-0.10^∗∗∗^	[-0.15, -0.05]
Partner	-0.03	[-0.15, 0.09]	0.90	[0.74, 1.09]	-0.09^∗∗∗^	[-0.15, -0.04]
**Control variables**						
**Education**						
Patient					-0.01	[-0.02, 0.01]
Partner					0.02^∗∗^	[0.01, 0.04]
**W.C. per 50 s**						
Patient	0.01^∗∗∗^	[0.01, 0.01]	1.01^∗∗∗^	[1.01, 1.02]	0.01^∗∗∗^	[0.01, 0.01]
Partner	0.02^∗∗∗^	[0.01, 0.02]	1.02^∗∗∗^	[1.01, 1.02]	0.01^∗∗∗^	[0.01, 0.01]
**Random effects**						
**Intercept (SD)**						
Patient	0.09		0.10		0.19	
Partner	0.10		0.13		0.15	
Residual (SD)	0.45		1.61		0.53	
Pseudo R-squared	21.27%		19.47%		27.61%	
-2log likelihood	18248.1		18564.53		9576.322	


*Est., Estimated result; Exp.(), Exponential results; CI, Confidence interval; WP, Within-person effects; Multiparty, Multiparty conversations; Education, Years of education; and W.C. per 50 s, Word count per 50 s. ^∗^*p* < 0.05, ^∗∗^*p* < 0.01, ^∗∗∗^*p* < 0.001.*

#### Unique Words: Entropy

As shown in Model 1 in Table 3, there was no main age effect on usage of unique words. Next, we found that patients reduced unique words when talking with children (*M*_patient_ = -0.26, *p* < 0.05) and friends (*M*_patient_ = -0.24, *p* < 0.01), whereas the effects for partners were non-significant. In order to test whether there were significant differences between patients and partners in the usage of unique words when talking with children and friends, we estimated a single-entry model with Child × Role interaction and Friend × Role interaction. The Child × Role interaction was non-significant (*b* = -0.12, *p* = 0.10, 95% CI [-0.27, 0.02]). That is, both patients and partners reduced unique words when talking with children. The Friend × Role interaction was significant (*b* = -0.14, *p* < 0.01, 95% CI [-0.25, -0.03]), which indicated that the effect of friends was significant for patients, but not for partners.

In line with our third research goal, we added Age × Interlocutor interactions to Model 1 to explore whether within-person interlocutor effects influence age effects in the usage of unique words. Although the effects for patients were not significant (*M*_patient_ = 0.00, *p* = 0.31, 95% CI [-0.02, 0.03]), we found that older partners uttered fewer unique words (*M*_partner_ = -0.02, *p* < 0.05, 95% CI [-0.04, 0.00]) with children than young partners. In order to test whether there were significant differences between patients and partners, we estimated a single-entry model with Child × Age × Role interaction. The three-way interaction was non-significant (*b* = 0.01, *p* = 0.10, 95% CI [0.00, 0.03]. Hence, older patients and partners used fewer unique words with children than younger patients and partners.

#### Uncommon Words: Average Frequency of Nouns

As depicted in Model 2 in Table 3, there were no main effects of age in the usage of uncommon words. Moreover, we did not find any interlocutor effects in the usage of uncommon words. These findings were contrary to our expectations. For the third research goal, we added Age × Interlocutor interactions to Model 2. Although Age × Children interaction in patients was non-significant (*b* = 1.01, *p* = 0.08, 95% CI [0.97, 1.05]), we found that young partners used more uncommon words than older partners when talking with children (*b* = 1.04, *p* < 0.05, 95% CI [1.01, 1.07]). Additionally, we found that young patients used more uncommon words than older patients when talking with friends [*b* = 1.02, *p* < 0.05, 95% CI (1.00, 1.04)]. The effects in partners were not significant [*b* = 1.00, *p* = 0.08, 95% CI (0.98, 1.02)]. In order to test whether there were significant differences between partners and patients in the aforementioned effects, we estimated a single-entry model with three-way interactions: Age × Children × Role and Age × Friends × Role. The interactions were non-significant (*bs* > 0.98, *ps* > 0.10, 95% CIs [0.96, 1.01] [1.00, 1.02]). That is, age differences existed in both patients and partners when talking with friends and children.

#### Grammatical Complexity: Clauses per Sentence

As presented in Model 3 in Table 3, there was no main effect of age in the usage of clauses per sentence. We found that compared to talking with their spouse, patients decreased 0.19 (*p* < 0.05) clauses per sentence with children and decreased 0.23 (*p* < 0.05) clauses per sentence with strangers. We found no significant results in partners when talking with children or strangers. Moreover, we found both patients and partners reduced their clauses per sentence in multiparty conversations (*M*_patient_ = -0.10, *M*_partner_ = -0.09, *p*s < 0.05). Next, we estimated a single-entry model with two-way interactions to test whether effects in children and strangers were significantly different between patients and partners. We found significant effects in Children × Role interaction (*b* = -0.07, *p* < 0.05, 95% CI [-0.14, 0.00]). That is, only patients decreased clauses per sentence with children. Furthermore, we found that the Stranger × Role interaction was non-significant (*b* = -0.09, *p* = 0.17, 95% CI = [-0.21, 0.04]). That is, both patients and partners reduced clauses per sentence when talking with strangers in comparison to talking with the spouse.

Finally, in line with our third research goal, Age × Interlocutor interactions were added to Model 3. We found that whereas the interaction Strangers × Age was not significant for patients (*M*_patient_ = 0.01, *p* = 0.07, 95% CI [0.00, 0.03]), older partners had fewer clauses per sentence (*M*_partner_ = -0.02, *p* < 0.05, 95% CI [-0.03, 0.00]) than young partners when talking with strangers. We, then, estimated a single-entry model and tested Age × Strangers × Role interaction and found a significant result (*b* = 0.01, *p* < 0.01, 95% CI [0.00, 0.02]). That is, the age differences in clauses per sentence when talking with strangers existed only in partners. In summary, Table 4 presents an overview of all significant fixed effects mentioned above.

**Table 4:** Summary of significant fixed effects.

Variable	Age	Within-person interlocutors	Age × Within-person interlocutors
Entropy		Child < Spouse ^a^For patients: Friend < Spouse	Decreased with age when talking to children
The average frequency of nouns			Increased with age when talking to children and friends
Clauses per sentence		Stranger < Spouse Multiparty < Spouse ^a^For patients: Child < Spouse	^b^For partners: Decreased with age when talking to strangers


*<, >, Comparisons indicated direction of significant effects; Multiparty, Multiparty conversations; ^*a*^For patients, Effects were only significant for patients; and ^*b*^For partners, Effects were only significant for partners.*

#### Control Variables

The effect of education was significant in clauses per sentence. Partners with higher education uttered more clauses per sentence (*M*_partner_ = 0.02, *p* < 0.01). In a single-entry model, we found a significant difference between patients and partners in the education effects (*b* = -0.01, *p* < 0.001, 95% CI [-0.02, -0.01]): Education had an effect on only partners’ clauses per sentence. Furthermore, a higher word count per 50 s was associated with more unique words, more common words, and more clauses per sentence.

## Discussion

This study, for the first time, examined age effects and within-person variations in real-life language use across interlocutors via a naturalistic observation method. Our results showed no overall age effects in the usage of unique words, uncommon words, and grammatical complexity when interlocutors were not taken into account. Compared to talking with their spouse, participants used fewer unique words with children and friends. Additionally, they used simpler grammatical structures with children, strangers, and in multiparty conversations. Next, we found that interlocutors influenced age effects in language use. More specifically, young adults used more unique words and more uncommon words with children than older adults. They used more uncommon words with friends and uttered more complex grammatical structures with strangers than older adults. Although it is not within the scope of our paper, we found some differences between patients and partners that do not affect the general pattern of findings.

### Age Effects and Real-Life Language Use

Contrary to past laboratory evidence which showed associations between language use and cognitive aging (Cheung and Kemper, 1992; Kavé et al., 2009), we did not find age effects in the usage of unique words, uncommon words, and grammatical complexity when interlocutors were not taken into account. Figure 2 shows that there are no obvious age effects on the usage of unique words and uncommon words. Although grammatical complexity seems to have higher scores in middle age, the age effect did not reach a significant level. Note that we also tested for quadratic age effects, but they were non-significant (*M*s = 0.00, *ps* > 0.05, 95% CIs [0.00, 0.00]). Horton et al. (2010) suggested that age effects were likely to be masked in real life, where speakers were allowed to use various ways to achieve their communication goals. In our preliminary analyses, we found that about 90% of the words in each 50-s sound file were within the most frequently used 2,000 words in the American National Corpus. Five-year-old children beginning school have a vocabulary of around 4,000 to 5,000 word families and university graduates have around 20,000 (Nation and Waring, 1997)^6^. This indicates that even if one has a rich vocabulary, they tend to use only a limited range of vocabulary in everyday life. Similarly, participants produced about 1.4 clauses per sentence in laboratory monologs (Kemper et al., 1989). In contrast, the number of clauses per sentence in this study ranged from 1.1 to 8 clauses per sentence, with less structured speech than speech in the laboratory^7^. Thus, our observations showed that language use in real life did not often represent individuals’ maximum level of verbal abilities that were tested in a uniform and stable environment such as the laboratory.

In theory, actual behavior is conceptualized as the interactions between personal characteristics and different supporting or impeding contexts (Diehl and Willis, 2003; WHO, 2015). Unlike the laboratory, where the upper limits of one’s abilities are tested (Baltes et al., 1984), in everyday life tasks, even though the upper limits of verbal abilities change with age, age effects can be attenuated when contextual demands are not high and when individuals can actively regulate their activities (Lewin, 1951; Martin and Moor, 2012). For example, in real life conversations, older adults may restrict themselves in certain occasions to a relatively limited vocabulary to foster communicative fluency or to avoid retrieval failures, such as tip-of-tongue experiences (James and Burke, 2000). Or young participants might produce simpler sentences in free conversations than in laboratory experiments that were designed to assess their verbal abilities (Cheung and Kemper, 1992). Such cases are likely to mask age effects in real-life language use. This proposition also fits with empirical findings which showed that cognitive decline in aging had a subtler impact on real-life cognitive activities than on cognitive performance in controlled laboratory settings (Park and Gutchess, 2000; Howieson, 2015).

Nevertheless, although the age range of our sample was between 24 and 94, most participants were between late 30 s and early 70 s. The small number of young participants in our sample may also explain the non-significant age effects in language use. In general, we consider our findings as preliminary evidence, as this study is the first attempt to use the EAR method to examine real-life language use in relation to age and within-person interlocutor effects. However, we believe our preliminary findings offer a new perspective for examining real-life language use.

### Within-Person Variations in Language Use Across Interlocutors

We found within-person variations in language use across interlocutors. These effects offered preliminary support for our perspective that interlocutors should be taken into account in real-life language use research. According to audience design research, the variations can be interpreted as individuals designing their language primarily in response to their audience (Horton and Gerrig, 2002; Schober and Brennan, 2003). We found that both patients and partners used fewer unique words with children. This observation is in line with the findings of Adams et al. (2002), which showed that participants used more repetitive words with a child than an experimenter. Repetition can form a rhythmic pattern that draws the audience’s attention (Tannen, 1987). Participants may have used repetition to help children to comprehend. Furthermore, patients used simpler grammatical structures with children than with the spouses. It is likely that patients used simpler sentences to help children to comprehend information, which is also in line with Adams et al. (2002). Nevertheless, fathers and mothers seemed to have different communicative styles when talking to their own children (e.g., Rowe et al., 2004) and gender differences in child-directed speech may have reflected onto grammatical complexity.

Furthermore, we found that patients used fewer unique words with friends than with the spouse. This finding is surprising: Rauers et al. (2011) showed that participants used fewer words with the spouse than with strangers, because familiarity with the audience allowed communication with fewer words. Accordingly, we reasoned our participants used more unique words with friends than with the spouse, particularly because the couples in our study were living together and knew each other well. We have a speculative explanation: The length of couples’ relationships ranged from 0.4 to 61.7 years and the degree of familiarity was likely to differ between couples. Long-term close friends can be more familiar than the spouse to some of our participants. Additionally, we note that the effect of friends on the usage of unique words was significant for patients, but not for partners. The difference may be due to gender differences in communication goals: Whereas men aim to establish and maintain status in conversations, women tend to engage in conversations to create and foster an intimate bond (Tannen, 1990). We cannot verify these speculations. However, they indicate an important issue for studying interlocutor effects in real-life language use, which we will discuss in the following paragraphs.

Moreover, we found both patients and partners used simpler grammatical structures when talking with strangers and in multiparty conversations than when talking with the spouse. Speakers were likely to reduce grammatical complexity to enhance the comprehension of the interlocutors who have less knowledge of the conversations (Rauers et al., 2011; Yoon and Brown-Schmidt, 2017). Our preliminary analysis showed that about 93% of the multiparty conversations included the spouse. Thus, participants using more unique words and fewer clauses per sentence in multiparty conversations than with their spouse are likely to engage in speech accommodation for other interlocutors in the group (except for the spouse), including friends (48%), children (33%), family members (22%), strangers (9%), and unknown people (4%).

Furthermore, we did not find differences between talking with family members in comparison to talking with the spouse. The differences in language use between the spouse and family members might be small, as they are all adult family members. Additionally, we did not find any differences in the usage of uncommon words across different interlocutors. The frequency of nouns seemed stable across interlocutors. This finding is in line with a past EAR study, which showed vocabulary usage to be stable across contexts (e.g., locations, activity) in young adults (Mehl and Pennebaker, 2003).

In sum, our findings demonstrated that the usage of unique words varied when talking with children and friends, and that grammatical complexity varied across children, strangers, and in multiparty conversations. Referring to past research on audience design, we offered multiple speculative explanations for the observed interlocutor effects. We cannot verify our speculations in this study, but these speculations did highlight one important issue: In real life, a certain type of interlocutor can entail complex and multidimensional impacts on language use, such as familiarity (Rauers et al., 2011) and needs of comprehension (Adams et al., 2002). Unlike in rigorous laboratory experiments with clear communication goals and tasks (Horton and Gerrig, 2016; Ferreira, 2019), the categorization of interlocutor types may not be adequate to fully explain the variations in usage of unique words, uncommon words, and grammatical complexity in real life. However, finding a significant fixed interlocutor effect is the first step toward establishing a new perspective of understanding real-life language use in the context of cognitive aging. As environments are more varied and unstable in real life than in the laboratory, we think it necessary to take into account contextual effects. The next step will be to investigate, in detail, the effects of context on real-life language use.

It is important to note that we have no intention of claiming that our findings confirm or disconfirm past research on audience design. We used past findings as a reference point to find potential explanations to interpret the observed interlocutor effects. To examine the mechanism of communication processes of audience design will require rigorous experimental designs (Horton and Gerrig, 2016; Ferreira, 2019), which is beyond the scope of this paper. Furthermore, the naturalistic observation approach prevents us from knowing speakers’ intentions in language variations across interlocutors. Future studies can benefit from our real-life observations and use a multi-method approach with experience sampling to obtain self-reports from participants.

### The Impact of Interlocutors on Age Effects in Language Use

In this study, we explored whether age effects in language use differed across interlocutors. The observed significant Age × Interlocutor interactions offered support for our anticipation that age effects on language use would be influenced by interlocutors. First, young adults used more unique words and more uncommon words than older adults with children. This finding is in line with laboratory observations where older adults used simpler language with children than young adults (Adams et al., 2002). According to the socioemotional selectivity theory (Carstensen et al., 1999), the emotional gains of social interaction take on greater importance than knowledge gains as one gets older. In turn, older adults may have used simpler language features (i.e., more repetitions and fewer uncommon words) to approach emotional gains in interactions with children. Second, young adults used more uncommon words than older adults when talking with friends. As knowledge gains are more important than emotional gains for young than older adults (Carstensen et al., 1999), it is likely that young adults exchange more diverse information in conversations with friends than older adults.

Finally, older partners used simpler grammatical structures with strangers than young partners. On the one hand, this finding can be interpreted as older adults simplifying their language more than young adults to convey information to strangers. Alternatively, conversations with strangers may involve new information, which imposes cognitive challenges and forces participants to reduce grammatical complexity, and older adults may have been affected more than young adults (Kemper et al., 2010). However, the age effect in grammatical complexity when talking with strangers was not significant for patients. Moscoso del Prado Martín (2016) found that grammatical complexity decreased in men in older age, but not in women. Gender differences may explain why we found age differences in only partners in grammatical complexity.

In real life where contexts are more diverse and unstable than in the laboratory, age effects in language use are evident in only some contexts, i.e., while talking to friends, children, and strangers. These contexts can represent different goal stimuli or cognitive challenges. Although we cannot confirm which mechanism caused the age differences, our findings suggest that effects of cognitive aging should not be examined in isolation from contexts. As there is growing interest in collecting “big data” and understanding cognitive activities in real life (e.g., Verhaeghen et al., 2012; Demiray et al., 2017, 2018), it should be useful to adopt the perspective of considering both individual characteristics and contexts.

### Education and Role (Gender) Effects on Language Use

Finally, we controlled for education, role, depression scores, and patients’ illness stage. First, partners with higher education produced more complex grammatical structures than partners with lower education. These findings were in line with past studies which examined telephone conversations and found educational effects in grammatical complexity Moscoso del Prado Martín (2016). However, we are not certain why the educational effects in grammatical complexity were not replicated with patients. Furthermore, unlike past studies (e.g., Meylan and Gahl, 2014), we did not find relations between the usage of unique and uncommon words, and education. In fact, our preliminary analysis showed that about 90% of uttered words were among the 2,000 most frequently used words, which might indicate that educational level is not relevant for real-life vocabulary.

Furthermore, as we used speech samples from breast cancer patients and their spouses, we found some differences between patients and partners: Some results were significant in only one group, but not the other. We assumed that these differences were gender differences, because men and women tend to have different conversational styles and prefer different conversational topics (Tannen, 1990; Pasupathi, 2003; Newman et al., 2008). However, without sufficient empirical evidence, we can only offer speculative explanations. Note that we have controlled for participants’ depression scores and patients’ cancer stage, and can exclude the possibility that these factors influence language use. Moreover, cancer was mentioned in only 5% of the couples’ conversations in this study (Robbins et al., 2014). Thus, we reasoned that having cancer did not have a big impact on the language use of our participants. Additionally, although some results were not consistently significant across patients and partners, we did not show any opposite patterns for the two groups. Thus, we believe these differences did not influence the general pattern of our findings.

### Limitations and Future Directions

This study contributed to the literature with its naturalistic observation method and adult lifespan sample. However, as it made the first attempt at examining age and within-person interlocutor effects in real-life language use, it had several limitations. First, some expected interlocutor effects were not observed in our sample, which can be due to the small number of observations for some interlocutors (e.g., family members). It is a limitation of naturalistic observation studies that some events/behaviors can occur infrequently and, thus, influence statistical estimations. Future studies could consider prolonging the data collection period to obtain sufficient observations for each interlocutor. Second, the reasons for language variation across different interlocutors in real life were unclear. We observed that language use varied across different interlocutors, but speakers’ actual intentions were unknown. Future studies should try to understand the subjective perceptions of speakers during language use. Combining our naturalistic observation method with experience sampling methodology (i.e., simultaneous collection of self-report from participants) would result in a stronger multi-method approach. Third, due to the complexity of our statistical models, we could not estimate the random effects of different interlocutors on language use. We thought it important to examine whether language use varied across different real-life contexts, before diving into individual differences in specific interlocutor effects. Future studies should investigate individual differences in interlocutor effects. Fourth, participants in this study were couples coping with breast cancer. Although we found that patients’ cancer stage and participants’ depression scores did not influence language use, patients may still have different communicative behaviors than healthy adults. Future studies should examine different samples to improve the understanding of real-life language use. However, we believe that our findings provide important preliminary insights about within-person variations in real-life language use. Finally, as gender (i.e., men, women) and role (i.e., partner, patient) were almost completely overlapping in this study, we were unable to empirically separate whether the differences in patients and partners were due to gender differences or role differences. Future studies should take into account gender effects in real-life language use and aging.

## Conclusion

Our naturalistic observation method is novel and has been used for the first time to understand age effects and within-person variations in language use in cognitive aging research. Thanks to this method, we were able to observe how adults varying in age actually speak in their natural environments. Motivated by the expectation that cognitive and contextual factors may interact to affect language use, we found that the usage of unique words and grammatical complexity varied when talking with different interlocutors (i.e., children, friends, strangers, in multiparty conversations). Additionally, age effects on language use were influenced by interlocutors (i.e., children, friends, strangers). In conclusion, this study, using a naturalistic observation method, contributes to the literature by examining the effects of age and within-person variations across interlocutors in real-life language use. Given its pioneering efforts, our results are preliminary. However, they offer a new perspective for examining real-life language use in the context of cognitive aging, with a focus on individual characteristics (i.e., age), context (i.e., interlocutors), and the interaction between individual characteristics and context.

## Ethics Statement

All study procedures were approved by the Institutional Review Board at the University of Arizona. All subjects gave written informed consent in accordance with the Declaration of Helsinki.

## Author Contributions

ML, BD, and MM developed the research concept and design. MR conducted the study as part of a larger project. ML conducted the data analyses and drafted the manuscript. ML and BD worked together on the interpretation of results and on framing and writing the manuscript. MM provided critical revisions on the manuscript.

## Conflict of Interest Statement

The authors declare that the research was conducted in the absence of any commercial or financial relationships that could be construed as a potential conflict of interest.

## Footnotes

1 The open source code of the Chao-Shen entropy estimator in the “entropy” package: https://github.com/cran/entropy/blob/master/R/entropy.ChaoShen.R.

2 Stranger(s) referred to the interlocutor who was clearly not an acquaintance of the participant. Unknown indicated the interlocutor whose relationship with the participant was unclear to the coders.

3 The comparably low ICC [1;2] of 0.51 came from talking with family members in patients. It was sometimes confusing to separate family members and friends of patients. Note that the ICC [1;2] of family members in partners were 0.86. The ICC [1;2] of strangers were 0.71, and the ICCs [1;2] of spouse, children, and friends were above 0.80.

4 We excluded two cases which had 10.50 and 20.00 clauses per sentence, because the high number of clauses was likely caused by missing punctuation in the transcripts.

5 Estimating complex random-effects models (i.e., random slopes) for a categorical variable with a large number of levels leads to a singularity problem and failure to converge. The binary variables of interlocutors in our study are equivalent to a categorical variable with multiple numbers of levels. Source: https://bbolker.github.io/mixedmodels-misc/glmmFAQ.html.

6 Word families are groups of words that have a common feature or pattern, e.g., the same combinations of letters and a similar sound. For example, at, cat, hat, and fat are a word family. Source: http://www.enchantedlearning.com/rhymes/wordfamilies/.

7 An example: “She’s kind of still having trouble with her father’s death but, and her sister told her maybe because she’s alone and the other siblings are together that it’s, they thought that it’s being harder on xxxx than it is on them.”
